# Contributions of histone tail clipping and acetylation in nucleosome transcription by RNA polymerase II

**DOI:** 10.1093/nar/gkad754

**Published:** 2023-09-18

**Authors:** Takumi Oishi, Suguru Hatazawa, Tomoya Kujirai, Junko Kato, Yuki Kobayashi, Mitsuo Ogasawara, Munetaka Akatsu, Haruhiko Ehara, Shun-ichi Sekine, Gosuke Hayashi, Yoshimasa Takizawa, Hitoshi Kurumizaka

**Affiliations:** Laboratory of Chromatin Structure and Function, Institute for Quantitative Biosciences, The University of Tokyo, 1-1-1 Yayoi, Bunkyo-ku, Tokyo 113-0032, Japan; Department of Biological Sciences, Graduate School of Science, The University of Tokyo, 1-1-1 Yayoi, Bunkyo-ku, Tokyo 113-0032, Japan; Laboratory of Chromatin Structure and Function, Institute for Quantitative Biosciences, The University of Tokyo, 1-1-1 Yayoi, Bunkyo-ku, Tokyo 113-0032, Japan; Laboratory of Chromatin Structure and Function, Institute for Quantitative Biosciences, The University of Tokyo, 1-1-1 Yayoi, Bunkyo-ku, Tokyo 113-0032, Japan; Laboratory for Transcription Structural Biology, RIKEN Center for Biosystems Dynamics Research, 1-7-22 Suehiro-cho, Tsurumi-ku, Yokohama 230-0045, Japan; Laboratory of Chromatin Structure and Function, Institute for Quantitative Biosciences, The University of Tokyo, 1-1-1 Yayoi, Bunkyo-ku, Tokyo 113-0032, Japan; Laboratory of Chromatin Structure and Function, Institute for Quantitative Biosciences, The University of Tokyo, 1-1-1 Yayoi, Bunkyo-ku, Tokyo 113-0032, Japan; Laboratory of Chromatin Structure and Function, Institute for Quantitative Biosciences, The University of Tokyo, 1-1-1 Yayoi, Bunkyo-ku, Tokyo 113-0032, Japan; Laboratory of Chromatin Structure and Function, Institute for Quantitative Biosciences, The University of Tokyo, 1-1-1 Yayoi, Bunkyo-ku, Tokyo 113-0032, Japan; Department of Biological Sciences, Graduate School of Science, The University of Tokyo, 1-1-1 Yayoi, Bunkyo-ku, Tokyo 113-0032, Japan; Laboratory for Transcription Structural Biology, RIKEN Center for Biosystems Dynamics Research, 1-7-22 Suehiro-cho, Tsurumi-ku, Yokohama 230-0045, Japan; Laboratory for Transcription Structural Biology, RIKEN Center for Biosystems Dynamics Research, 1-7-22 Suehiro-cho, Tsurumi-ku, Yokohama 230-0045, Japan; Department of Biomolecular Engineering, Graduate School of Engineering, Nagoya University, Furo-cho, Chikusa-ku, Nagoya 464-8603, Japan; Laboratory of Chromatin Structure and Function, Institute for Quantitative Biosciences, The University of Tokyo, 1-1-1 Yayoi, Bunkyo-ku, Tokyo 113-0032, Japan; Laboratory of Chromatin Structure and Function, Institute for Quantitative Biosciences, The University of Tokyo, 1-1-1 Yayoi, Bunkyo-ku, Tokyo 113-0032, Japan; Department of Biological Sciences, Graduate School of Science, The University of Tokyo, 1-1-1 Yayoi, Bunkyo-ku, Tokyo 113-0032, Japan; Laboratory for Transcription Structural Biology, RIKEN Center for Biosystems Dynamics Research, 1-7-22 Suehiro-cho, Tsurumi-ku, Yokohama 230-0045, Japan

## Abstract

The N-terminal tails of histones protrude from the nucleosome core and are target sites for histone modifications, such as acetylation and methylation. Histone acetylation is considered to enhance transcription in chromatin. However, the contribution of the histone N-terminal tail to the nucleosome transcription by RNA polymerase II (RNAPII) has not been clarified. In the present study, we reconstituted nucleosomes lacking the N-terminal tail of each histone, H2A, H2B, H3 or H4, and performed RNAPII transcription assays. We found that the N-terminal tail of H3, but not H2A, H2B and H4, functions in RNAPII pausing at the SHL(-5) position of the nucleosome. Consistently, the RNAPII transcription assay also revealed that the nucleosome containing N-terminally acetylated H3 drastically alleviates RNAPII pausing at the SHL(-5) position. In addition, the H3 acetylated nucleosome produced increased amounts of the run-off transcript. These results provide important evidence that the H3 N-terminal tail plays a role in RNAPII pausing at the SHL(-5) position of the nucleosome, and its acetylation directly alleviates this nucleosome barrier.

## Graphical Abstract

**Graphical Abstract:**
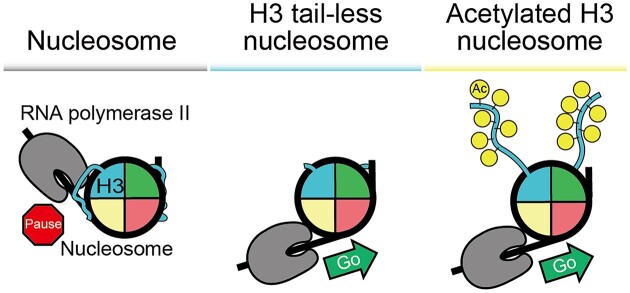

## INTRODUCTION

Histones H2A, H2B, H3 and H4 are the protein components of the nucleosome, an elemental structural unit of eukaryotic chromatin (1). Histones are composed of intrinsically disordered N- and/or C-terminal tails and histone-fold domains (2,3). The H2A–H2B and H3–H4 heterodimers are formed by interactions between the histone-fold domains, and the histone octamer is established with two each of the H2A–H2B and H3–H4 dimers (2). The nucleosome core is composed of the histone octamer and approximately 150 bp of DNA, which is left-handedly wrapped around it in a symmetrical manner (4). The nucleosomal DNA positions are designated as superhelical locations (SHLs) (4,5). The center of the nucleosomal DNA is SHL(0), which corresponds to the symmetry axis of the nucleosome structure (4,5). The nucleosomal DNA positions about every 10 base pairs from the SHL(0) position are referred to as SHL(±1), SHL(±2), SHL(±3), SHL(±4), SHL(±5), SHL(±6) and SHL(±7). In the nucleosome, the N-terminal tails of histones project out from the DNA superhelical gyres (6).

The histone N-terminal tails contain many basic residues, such as Lys and Arg (6). The Lys residues of the histone N-terminal tails are the major target sites for post-translational modifications (PTMs), such as acetylation and methylation, which are introduced by enzymatic ‘writers’, histone acetyltransferases and methyltransferases, respectively (7,8). Chromatin binding proteins called ‘readers’ specifically target these PTMs of the histone N-terminal tails, and are recruited to the genomic loci where they function in genome regulation (7,8). Accumulations of acetylated histones have been found in transcriptionally active loci in cells (9). In addition, the acetylation of all histones reportedly enhances transcription on chromatinized DNA templates *in vitro* (10–12). These findings suggest that the acetylation of histone tails generally facilitates transcription in chromatin.

The basic residues of histone tails may bind to the DNA within the nucleosome core and linkers (13). The Lys acetylation neutralizes its basic side chain, thus weakening the binding of histone tails to the nucleosomal DNA. In fact, nuclear magnetic resonance (NMR) analyses revealed that the acetylation of histone tails significantly increases the histone-tail dynamics in the nucleosome (14–16). Consistently, a fluorescence resonance energy transfer (FRET) analysis demonstrated that H3 N-terminal tail acetylation enhances the flexibility of nucleosomal DNA ends (17). The weakened DNA binding by the histone tail acetylation may enhance the accessibility of chromatin binding proteins and the processivity of DNA-dependent enzymes, such as RNA polymerases.

During transcription in the nucleosome, RNA polymerase II (RNAPII) gradually peels the DNA from the histone octamer and proceeds with major pauses at SHL(-5) and SHL(-1) (18), which are consistently found in genome-wide studies (19). These nucleosome barriers of RNAPII transcription are substantially alleviated by transcription elongation factors (20). The nucleosome is finally transferred from ahead to behind the transcribing RNAPII, with the aid of histone chaperones and transcription elongation factors (21). The histone core structures during these nucleosome transcription processes have been visualized by cryo-electron microscopy (cryo-EM). However, the contributions of histone N-terminal tails to nucleosome transcription have not been elucidated, due to their flexible nature.

To study the influences of histone N-terminal tails on RNAPII transcription in the nucleosome, we performed RNAPII transcription assays with nucleosomes containing N-terminally deleted histones. We found that the deletion of the N-terminal tail of H3, but not H2A, H2B and H4, alleviates the RNAPII pausing at the SHL(-5) position. We further studied RNAPII transcription in the nucleosome containing the N-terminally acetylated, full-length H3 protein, and determined that the acetylation of the H3 N-terminal tail drastically decreases the RNAPII pausing at the SHL(-5) position, and also increases the run-off transcript. These results suggest that the acetylation of the H3 N-terminal tail may directly alleviate the nucleosome barrier, and function as a regulator for RNAPII transcription elongation in chromatin.

## MATERIALS AND METHODS

### Preparation of histones

Human histones H2A, H2B, H3.1 and H4, and human tail-less histones H2A, H2B, H3.1 and H4 lacking N-terminal tails were produced in *Escherichia coli* cells and purified by the method described previously (22,23). The H3.2 C110A, H3K4/9/14Ac, H3K18/23/27Ac and H3K4/9/14/18/23/27Ac peptides were produced by the method described previously (17). In brief, the chemically synthesized H3 N-terminal peptides containing specific histone acetylation patterns were ligated with the recombinant C-terminal regions of H3 through a one-pot chemical ligation process, followed by desulfurization.

### Preparation of DNA fragments

The 145 bp 601 DNA was prepared as previously described (24,25). The palindromic derivative of the Widom 601 DNA, the 193 bp Widom 601L DNA, was generated and prepared as described (26,27). In brief, multiple repeats of each half of the 601L DNA were generated in the pGEM-T Easy vector. Each half of the 601L DNA was excised from the vector by digestion with EcoRV (Takara), followed by PEG precipitation to separate the vector DNA and the DNA fragment. The resulting DNA fragments were dephosphorylated by Calf Intestinal Alkaline Phosphatase (Takara), extracted by phenol–chloroform extraction, and precipitated with ethanol. DNA fragments were cleaved by HinfI (Takara) and purified through TSK-DEAE ion exchange chromatography. The purified DNA fragments were ligated, and unligated fragments were separated using a Prep Cell apparatus (Bio-Rad). The 193 bp Widom 601L DNA sequence is as follows: ATCACGTAATATTGGCCAGCTAGGATCACAATCCCGGTGCCGAGGCCGCTCAATTGGTCGTAGACAGCTCTAGCACCGCTTAAACGCACGTACGGAATCCGTACGTGCGTTTAAGCGGTGCTAGAGCTGTCTACGACCAATTGAGCGGCCTCGGCACCGGGATTGTGATCCTAGCTGGCCAATATTACGTGAT.

### Reconstitution and purification of nucleosomes

The histone octamers were prepared as previously described (22). The salt-dialysis method was used to reconstitute the 193 bp 601L canonical nucleosome, the 193 bp 601L tail-less nucleosome, the 145 bp 601 nucleosome containing the H3.2 C110A peptide, and the 145 bp 601 nucleosomes containing the acetylated H3.2 C110A peptide (22). The reconstituted nucleosomes were purified using a Prep Cell apparatus (Bio-Rad), followed by buffer exchange to 20 mM Tris–HCl (pH 7.5), 1 mM DTT and 5% glycerol.

### Purification of PL2-6 scFv^15^ and PL2-6 scFv^20^

In this study, we used PL2-6 scFv^15^ and PL2-6 scFv^20^, which contain (GGGGS)_3_ and (GGGGS)_4_ linker peptides between their heavy chain and light chain fragments, respectively. PL2-6 scFv^15^ was purified as previously described (28–30). The construction of the plasmid expressing PL2-6 scFv^20^ was reported previously (29). PL2-6 scFv^20^ was produced in the *Escherichia coli* BL21(DE3) strain by induction with 0.5 mM isopropyl β-d-1-thiogalactopyranoside. The harvested cells were disrupted by sonication. After centrifugation, the pellet was washed several times with wash buffer [50 mM Tris–HCl (pH 8.0), 100 mM NaCl, 1% Triton X-100 and 1 M urea]. After the final wash with phosphate-buffered saline, the pellet was dissolved in denaturing buffer [100 mM Tris and 6 M urea] prepared just before use, and stirred overnight. The supernatant was collected by centrifugation and mixed with nickel-nitrilotriacetic acid agarose resin. The protein-bound beads were washed with 25 column volumes of denaturing buffer containing 20 mM imidazole, mand eluted with denaturing buffer with 400 mM imidazole. The eluted sample was dialyzed against denaturing buffer, and the dialysis buffer was subsequently replaced with refolding buffer [50 mM Tris–HCl (pH 7.5), 150 mM NaCl and 1 mM EDTA] using a peristaltic pump (0.8 ml/min). The sample was then dialyzed against refolding buffer for at least 4 h. The refolded scFv was purified on a HiLoad 26/600 Superdex 75 pg (Cytiva) column equilibrated with refolding buffer, at a flow rate of 0.6 ml/min. Eluted fractions were analyzed by SDS-PAGE, with and without reductant in the SDS-PAGE sample buffer.

### Preparation of nucleosomes for cryo-EM analysis

The purified canonical nucleosome was mixed with PL2-6 scFv^15^ at a molar ratio of 1:6, in reaction buffer containing 10 mM Tris–HCl (pH 7.5), 30 mM NaCl, 1 mM DTT, 0.2 mM EDTA and 1% glycerol. After an incubation at 25°C for 30 min, the solution was subjected to plunge freezing.

For the tail-less nucleosome, the nucleosome was mixed with PL2-6 scFv^15^ at a 1:8 ratio, in reaction buffer containing 20 mM Tris–HCl (pH 7.5), 50 mM NaCl, 0.7 mM DTT, 0.3 mM EDTA and 3.3% glycerol. The solution was subjected to plunge freezing.

For the non-acetylated nucleosome and the nucleosome containing the H3K4/9/14/18/23/27Ac peptide, the nucleosome was mixed with PL2-6 scFv^20^ at a 1:2 ratio, in reaction buffer containing 21.8 mM Tris–HCl (pH 7.5), 30 mM NaCl, 0.6 mM DTT, 0.2 mM EDTA and 2.9% glycerol. After an incubation at 30°C for 30 min, the solution was utilized for plunge freezing.

All cryo-EM analysis samples were plunge frozen using a Vitrobot Mark IV (Thermo Fisher Scientific) on a Quantifoil R1.2/1.3 copper grid. The grid was glow discharged for 1 min, using a PIB-10 Bombarder (Vacuum Device Inc.).

### Cryo-EM data collection

Cryo-EM data were collected with the EPU automation software on a Krios G4 cryo-EM (Thermo Fisher Scientific). The cryo-EM was operated at 300 kV, with a nominal magnification of 81 000× (pixel size of 1.06 Å) and a defocus range of −1.0 to −2.5 μm. Micrographs of the nucleosomes were recorded with an exposure time of 4.5 s on a K3 BioQuantum direct detection camera (Gatan) in the energy-filter mode with 25 eV, at a total dose of ∼60 electrons/Å^2^, with a total of 40 frames.

### Image processing

In total, 7 012 movies for the canonical nucleosome, 4 665 movies for the tail-less nucleosome, 6 104 movies for the non-acetylated nucleosome, and 5 324 movies for the nucleosome containing the H3K4/9/14/18/23/27Ac peptide were aligned with MOTIONCOR2 (31), with dose weighting. The contrast transfer function (CTF) was estimated by CTFFIND4 from micrographs with dose weighting (32). Relion 3.1 or Relion 4.0 was used for the following image processing (33,34). The resolution of the final density map was estimated by the gold standard Fourier Shell Correlation at an FSC = 0.143 (35). The refined maps were postprocessed with DeepEMhancer (36). For the canonical nucleosome, 354 676 particles were automatically picked by LoG-based auto-picking from 820 micrographs. After removing junk particles by 2D classification, 23 373 particles were used as the reference to pick 291 681 particles from 820 micrographs. Subsequently, 226 681 particles were selected by 2D classification, and 97 708 particles were selected by the following 3D classification. The cryo-EM map of the 197 bp nucleosome with PL2-6 scFv (EMDB: EMD-22686 (37)) was used as the initial reference model. After the 3D refinement, the density map was used for Topaz (38) particle picking, and 3 086 435 particles were picked from 6 349 micrographs. In total, 1 938 540 particles were selected by 2D classification, and 594 121 particles were selected by 3D classification, followed by Bayesian polishing and CTF refinement. After 3D refinement and sharpening with a *B*-factor of −74.475 Å^2^, the resolution of the density map was estimated to be 2.65 Å.

For the tail-less nucleosome, 1 286 452 particles were automatically picked by LoG-based auto-picking from 3 898 micrographs. Next, 468 990 particles were selected by 2D classification, and 47 360 particles were selected by the following 3D classification. The cryo-EM map of the 197 bp nucleosome with PL2-6 scFv (EMDB: EMD-22686 (37)) was used as the initial reference model. After the 3D refinement, the density map was used for Topaz (38) particle picking, and 1 002 273 particles were picked from 3 898 micrographs. Subsequently, 620 873 particles were selected by 2D classification, and 113 058 particles were selected by 3D classification, followed by Bayesian polishing and CTF refinement. After 3D refinement and sharpening with a *B*-factor of −113.116 Å^2^, the resolution of the density map was estimated to be 3.44 Å.

For the non-acetylated nucleosome, 428 651 particles were picked from 500 micrographs, using the 147 bp nucleosome with PL2-6 scFv (EMDB: EMD-8938 (28)) as the reference model. Next, 349 199 particles were selected by 2D classification, and 144 481 particles were selected by the following 3D classification. After the 3D refinement, the density map was used for Topaz (38) particle picking, and 4 721 708 particles were picked from 6 025 micrographs. Subsequently, 3 687 400 particles were selected by 2D classification, and 1 757 905 particles were selected by 3D classification, followed by Bayesian polishing and CTF refinement. After 3D refinement and sharpening with a B-factor of −89.459 Å^2^, the resolution of the density map was estimated to be 2.36 Å.

For the nucleosome containing the H3K4/9/14/18/23/27Ac peptide, 737 492 particles were picked from 500 micrographs, using the 147 bp nucleosome with PL2-6 scFv (EMDB: EMD-8938 (28)) as the reference model. In total, 518 933 particles were selected by 2D classification, and 161 464 particles were selected by the following 3D classification. After the 3D refinement, the density map was used for Topaz (38) particle picking, and 4 338 139 particles were picked from 5 198 micrographs. Subsequently, 3 961 904 particles were selected by 2D classification, and 946 229 particles were selected by 3D classification, followed by Bayesian polishing and CTF refinement. After 3D refinement and sharpening with a B-factor of −93.890 Å^2^, the resolution of the density map was estimated to be 2.48 Å. The details of the processing statistics for the canonical nucleosome, the tail-less nucleosome, the non-acetylated nucleosome and the nucleosome containing the H3K4/9/14/18/23/27Ac peptide are provided in Table 1. Structural figures were rendered using UCSF ChimeraX (39).

**Table 1.** Cryo-EM data collection, processing, refinement and validation statistics

Sample	Canonical nucleosome	Tail-less nucleosome	H3.2 C110A nucleosome	H3 acetylated nucleosome
**Data collection**				
Electron microscope	KriosG4	KriosG4	KriosG4	KriosG4
Camera	K3	K3	K3	K3
Pixel size (Å/pix)	1.06	1.06	1.06	1.06
Defocus range (μm)	−1.0 to −2.5	−1.0 to −2.5	−1.0 to −2.5	−1.0 to −2.5
Exposure time (second)	4.5	4.5	4.5	4.5
Total dose (e^−^/Å^2^)	63	59	60	60
Movie frames (no.)	40	40	40	40
Total micrographs (no.)	7 012	4 665	6 104	5 324
**Reconstruction**				
Software	Relion 3.1	Relion 3.1	Relion 4.0	Relion 4.0
Particles for 2D classification	3 086 435	1 002 273	4 721 708	4 338 139
Particles for 3D classification	1 938 540	620 873	3 687 400	3 961 904
Particles in the final map (no.)	594 121	113 058	1 757 905	946 229
Symmetry	C1	C1	C1	C1
Final resolution (Å)	2.65	3.44	2.36	2.48
FSC threshold	0.143	0.143	0.143	0.143
Map sharpening **B** factor (Å^2^)	−74.475	−113.116	−89.459	−93.890
**Model building**				
Software	Coot	Coot	Coot	Coot
**Refinement**				
Software	Phenix, ISOLDE	Phenix, ISOLDE	Phenix	Phenix
**Model composition**				
Protein	753	748	746	746
Nucleotide	310	312	290	290
**Validation**				
MolProbity score	1.02	1.15	1.15	1.24
Clash score	2.36	3.59	3.57	4.69
R.m.s. deviations				
Bond lengths (Å)	0.006	0.006	0.004	0.004
Bond angles (°)	0.970	0.982	0.693	0.706
**Ramachandran plot**				
Favored (%)	98.10	98.63	98.49	98.63
Allowed (%)	1.90	1.37	1.51	1.37
Outliers (%)	0.00	0.00	0.00	0.00

### Model building

The atomic models of the 193 bp 601L canonical nucleosome and the tail-less nucleosome were built using the atomic coordinates of the 197 bp 601 human nucleosome (PDB ID: 7K61 (37)) and the 145 bp 601L human nucleosome (PDB ID: 7VZ4). Atomic coordinates of the nucleosomes were fitted to the cryo-EM maps by rigid body fitting with UCSF ChimeraX (39). The resulting atomic models were refined by Phenix real space refine (40) against the cryo-EM map, and edited manually with the COOT and ISOLDE software (41,42). The atomic models of the 145 bp 601 non-acetylated nucleosome and the acetylated nucleosome containing the H3K4/9/14/18/23/27Ac peptide were built using the atomic coordinates of the 145 bp 601 *Xenopus laevis* nucleosome (PDB ID: 7OHC (43)) and the 145 bp 601L human nucleosome (PDB ID: 7VZ4). Atomic coordinates of the nucleosomes were fitted to the cryo-EM map by rigid body fitting with UCSF ChimeraX (39). The resulting atomic models were refined by phenix real space refine (40) against the cryo-EM map, and edited manually with the COOT software (41).

### *In vitro* nucleosome transcription assay

A 153 bp DNA fragment containing a modified Widom 601 DNA sequence was ligated with a 45 bp DNA fragment with a 9 base mismatched region (18). The canonical nucleosome, the tail-less nucleosomes lacking each of the histone N-terminal tails, the non-acetylated H3.2 C110A nucleosome, and the acetylated nucleosomes containing the H3K4/9/14Ac, H3K18/23/27Ac and the H3K4/9/14/18/23/27Ac peptides were reconstituted by the salt dialysis method (22). The reconstituted nucleosomes were purified using a Prep Cell apparatus (Bio-Rad). *Komagataella pastoris* RNAPII and TFIIS were purified as described previously (18). The nucleosomes (0.1 μM) were transcribed at 30°C by RNAPII (0.1 μM), in buffer containing 0.1 μM TFIIS, 0.4 μM fluorescently labeled RNA primer (5′-DY647-AUAAUUAGCUC-3′) (Dharmacon), 26 mM HEPES–KOH (pH 7.5), 5 mM MgCl_2_, 50 mM potassium acetate, 0.2 μM zinc acetate, 20 μM Tris(2-carboxyethyl) phosphine, 0.1 mM DTT, 1.5% glycerol, 400 μM UTP, 400 μM CTP, 400 μM GTP and 400 μM ATP. The reaction was stopped by adding a deproteinization solution [200 mM Tris–HCl (pH 8.0), 80 mM EDTA, and 0.5 μg/μl proteinase K (Roche)]. The samples were mixed with Hi-Di formamide, incubated at 95°C for 10 min, and then analyzed by 10% denaturing-PAGE in 1× TBE buffer. An Amersham Typhoon imager (Cytiva) was used to detect the DY647 fluorescence of RNA products. The band intensities of the transcripts were quantified and normalized with those of the non-acetylated template transcripts, using the ImageQuant™ TL software (GE Healthcare).

## RESULTS

### Histone N-terminal tail removal does not affect the nucleosome core structure

**Figure 1.**
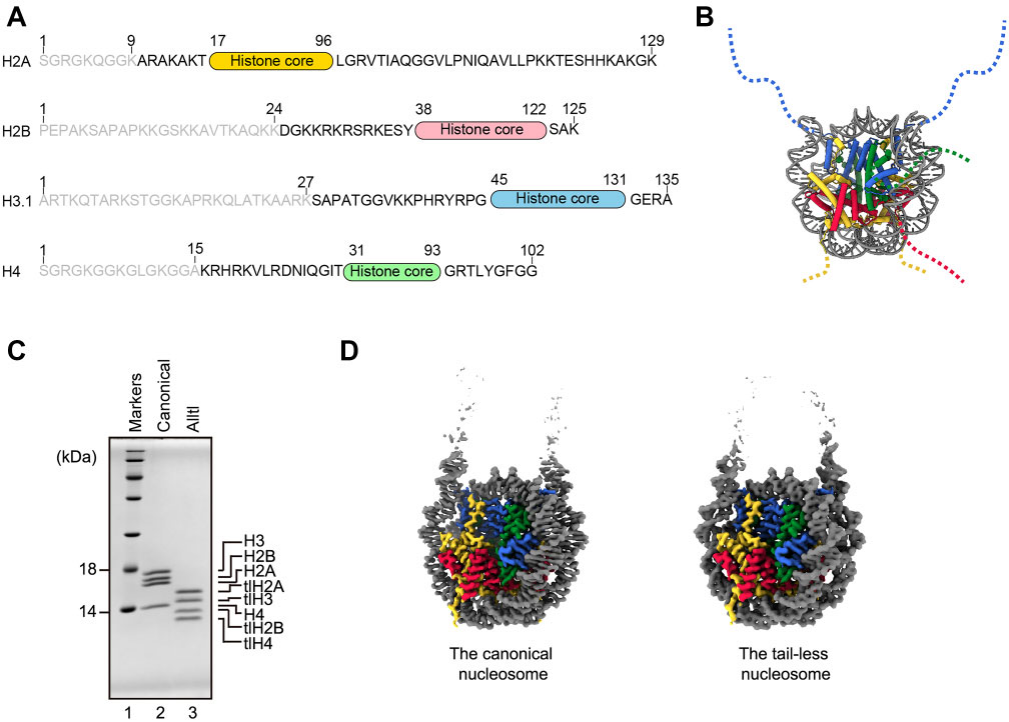
Cryo-EM structure of the nucleosome containing tail-less histones. (**A**) Amino acid sequences of N- and C-terminal regions of histones H2A, H2B, H3 and H4. Deleted regions of tail-less histones are shown in gray. (**B**) The schematic representation of the N-terminal regions of histones in the nucleosome structure. The unmodeled N-terminal regions of histones are shown in dotted lines on the human nucleosome structure (PDB ID: 7VZ4). DNA, histones H2A, H2B, H3, and H4 are colored gray, yellow, red, blue, and green, respectively. (**C**) The canonical nucleosome and tail-less nucleosome were analyzed by SDS-PAGE with CBB (Coomassie Brilliant Blue) staining. (**D**) Cryo-EM map of the canonical nucleosome and the tail-less nucleosome. DNA, histones H2A and tlH2A, H2B and tlH2B, H3 and tlH3, H4 and tlH4 are colored gray, yellow, red, blue, and green, respectively.

The N-terminal tails of histones provide major target sites for post-translational modifications, and play important roles in the regulation of genome function (8). The N-terminal tails of histones protrude from the nucleosome core (Figure 1B). We prepared the histone mutants named tail-less H2A (tlH2A), tail-less H2B (tlH2B), tail-less H3 (tlH3) and tail-less H4 (tlH4), in which the N-terminal tails of histones H2A, H2B, H3 and H4 were deleted, respectively (Figure 1A). These deleted regions of histones correspond to the major regions in the nucleosome removed by trypsin protease (44,45). The nucleosomes composed of these tail-less histones were reconstituted with a 193 base-pair DNA (Figure 1C). The cryo-EM structures of the nucleosomes composed of each tail-less histone H2A, H2B, H3, and H4 were determined by cryo-EM single-particle analysis (Figure 1D). The cryo-EM samples were prepared in the presence of the PL2-6 single-chain antibody variable fragment (scFv), which stabilizes the nucleosome without crosslinking (28,29) (Supplementary Figures S1 and S2). We previously demonstrated by X-ray crystallography that the deletion of each histone N-terminal tail did not alter the structure of the nucleosome (23). Interestingly, the simultaneous removal of all N-terminal histone tails did not affect the overall structure of the nucleosome core (Figure 1D). This fact assured the structural integrity of the nucleosomes containing the histone mutants lacking these N-terminal histone regions.

### The N-terminal tail of histone H3, but not histones H2A, H2B, and H4, suppresses transcription elongation by RNA polymerase II

**Figure 2.**
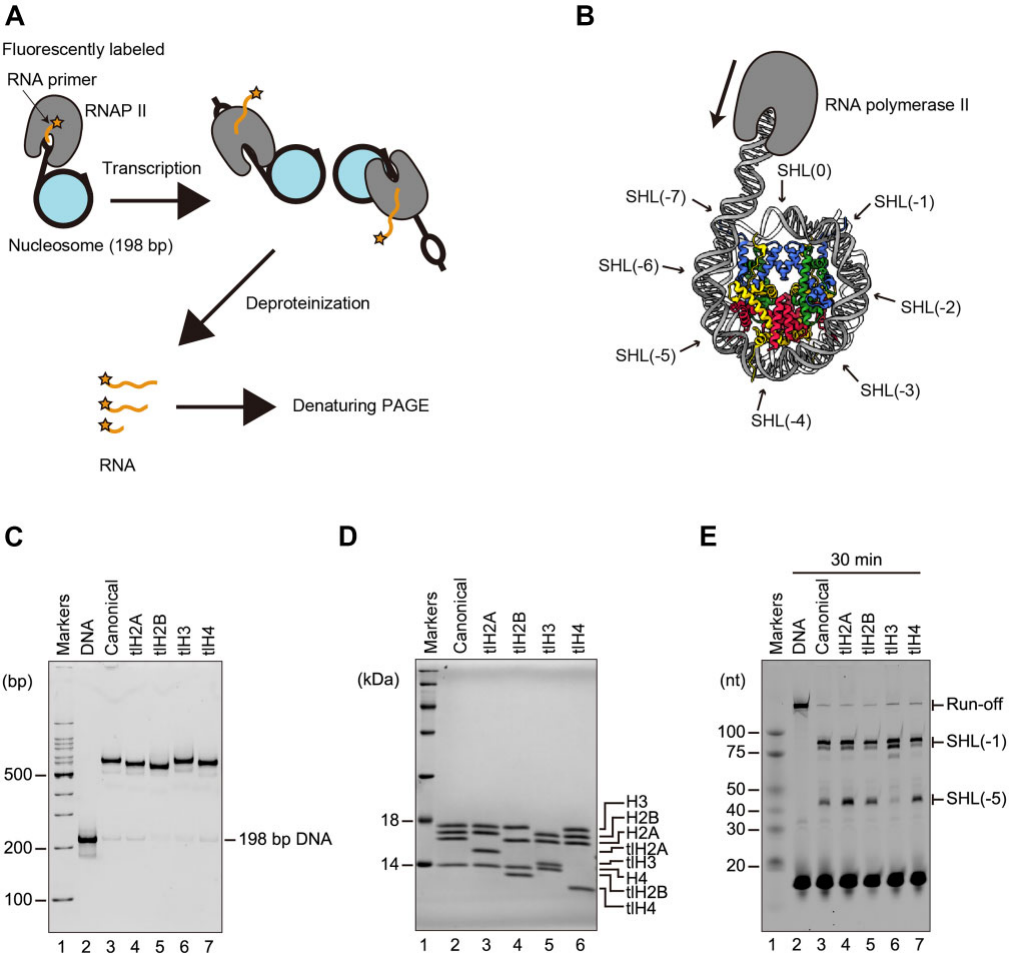
*In vitro* transcription assays of tail-less nucleosomes. (**A**) Schematic representation of the *in vitro* transcription assay. (**B**) SHLs and the direction of transcription by RNAPII are labeled on the cryo-EM structure of the human nucleosome (PDB ID: 7K61). DNA, histones H2A, H2B, H3, and H4 are colored gray, yellow, red, blue, and green, respectively. (**C**) The canonical nucleosome and the nucleosomes lacking each N-terminal histone tail were analyzed by nondenaturing-PAGE with ethidium bromide staining. A 198 bp DNA fragment was used for the nucleosome reconstitution. (**D**) Histone compositions of the canonical nucleosome and the nucleosomes lacking each N-terminal histone tail were analyzed by SDS-PAGE with CBB staining. (**E**) Transcription assays of the 198 bp DNA, the canonical nucleosome, and the nucleosomes lacking each N-terminal histone tail. RNA transcripts were analyzed by denaturing-PAGE, and detected by DY647 fluorescence. The reaction was conducted for 30 min. The transcription experiments were repeated three times, and the reproducibility was confirmed (Supplementary Figure S3).

To assess the contribution of each histone N-terminal tail to transcription, we performed the nucleosome transcription assay with RNAPII (Figure 2A). To do so, we reconstituted the nucleosomes containing tlH2A, tlH2B, tlH3 or tlH4 with the 198 base-pair DNA, in which a nine base-pair mismatch region is included in the linker DNA for the RNAPII transcription start site (Figure 2B–D). The transcription reaction was conducted from the linker DNA connected to the SHL(-7) side and proceeded toward the SHL(0) direction of the nucleosomal DNA, in the presence of the essential elongation factor TFIIS (Figure 2B).

When the transcription reaction was conducted with the naked DNA template, the full-length RNA product was detected as the run-off transcript (Figure 2E, lane 2). In contrast, the production of the run-off transcript was drastically decreased when the nucleosome was used as the template for RNAPII transcription (Figure 2E, lane 3). In agreement with the previous results (18,20), RNAPII transcription elongation was paused at the SHL(–5) and SHL(–1) positions of the nucleosomal DNA (Figure 2E, lane 3). We then performed the transcription assay with each nucleosome containing tlH2A, tlH2B, tlH3 or tlH4. The profiles of nucleosome transcription by RNAPII were not affected when the nucleosome containing tlH2A, tlH2B, or tlH4 was used (Figure 2E, lanes 4, 5 and 7). In contrast, the RNAPII pausing at the SHL(–5) position was substantially decreased when the transcription reaction was conducted with the nucleosome containing tlH3 (Figure 2E, lane 6). These results indicated that the N-terminal tail of H3 contributes to the RNAPII pausing at the SHL(–5) position of the nucleosome.

### Acetylation of the histone H3 N-terminal tail enhances transcription elongation by RNA polymerase II

**Figure 3.**
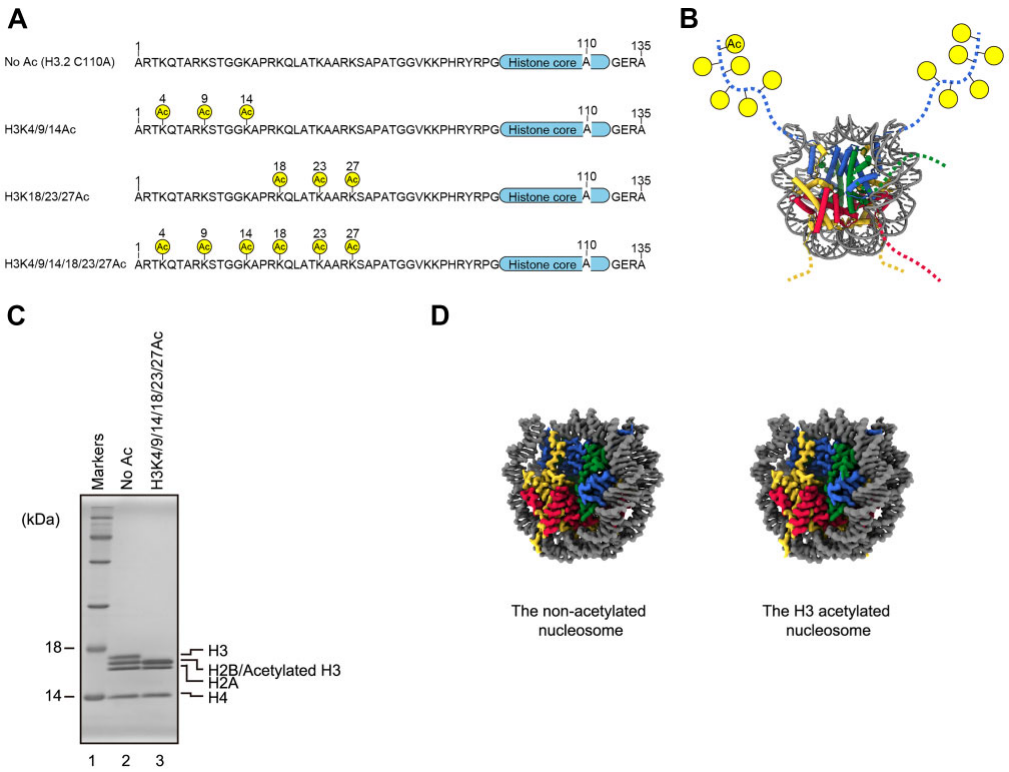
Cryo-EM structure of the nucleosome containing an H3 peptide with a highly acetylated N-terminal tail. (**A**) Amino acid sequences of the N-terminal regions of H3.2. The acetylated residues are labeled with yellow circles. (**B**) The schematic representation of the acetylated H3 tails in the nucleosome structure. The unmodelled N-terminal regions of histones and the acetylated residues (H3K4/9/14/18/23/27Ac) are shown in dotted lines and yellow circles, respectively, on the human nucleosome structure (PDB ID: 7VZ4). DNA, histones H2A, H2B, H3 and H4 are colored gray, yellow, red, blue, and green, respectively. (**C**) The non-acetylated nucleosome containing H3.2 C110A and the nucleosome containing the H3K4/9/14/18/23/27Ac peptide were analyzed by SDS-PAGE with CBB staining. (**D**) Cryo-EM structures of the nucleosome containing H3.2 C110A and the nucleosome containing the H3K4/9/14/18/23/27Ac peptide. DNA, histones H2A, H2B, H3.2 and H4 are colored gray, yellow, red, blue, and green, respectively.

Histone acetylation generally enhances RNAPII transcription (8,46). The tlH3 peptide used in the RNAPII transcription assay lacked the H3 Lys4, Lys9, Lys14, Lys18, Lys23 and Lys27 residues for acetylation (Figures 1 and 2) (23). We next prepared the full-length H3 peptide, H3K4/9/14/18/23/27Ac, in which an acetyl-lysine residue was inserted at these six positions, the Lys4, 9, 14, 18, 23 and 27 sites, by the chemical ligation method (Figure 3A and 3B). Since all of the Cys residues are converted into Ala residues during the desulfurization process after peptide ligation, we used the H3.2 C110A mutant, in which the Cys110 residue of H3.2 was replaced by Ala. There is only one amino acid difference, at position 96 (H3.1 Cys96 and H3.2 Ser96), between human H3.1 and H3.2. We then reconstituted the nucleosome containing H3K4/9/14/18/23/27Ac, and determined its cryo-EM structure (Figure 3C and D). Consistent with the structure of the all tail-less nucleosome (Figure 1D), the overall structure of the nucleosome containing acetylated H3 was quite similar to that of the non-acetylated nucleosome (Figure 3D, Supplementary Figures S4 and S5).

**Figure 4.**
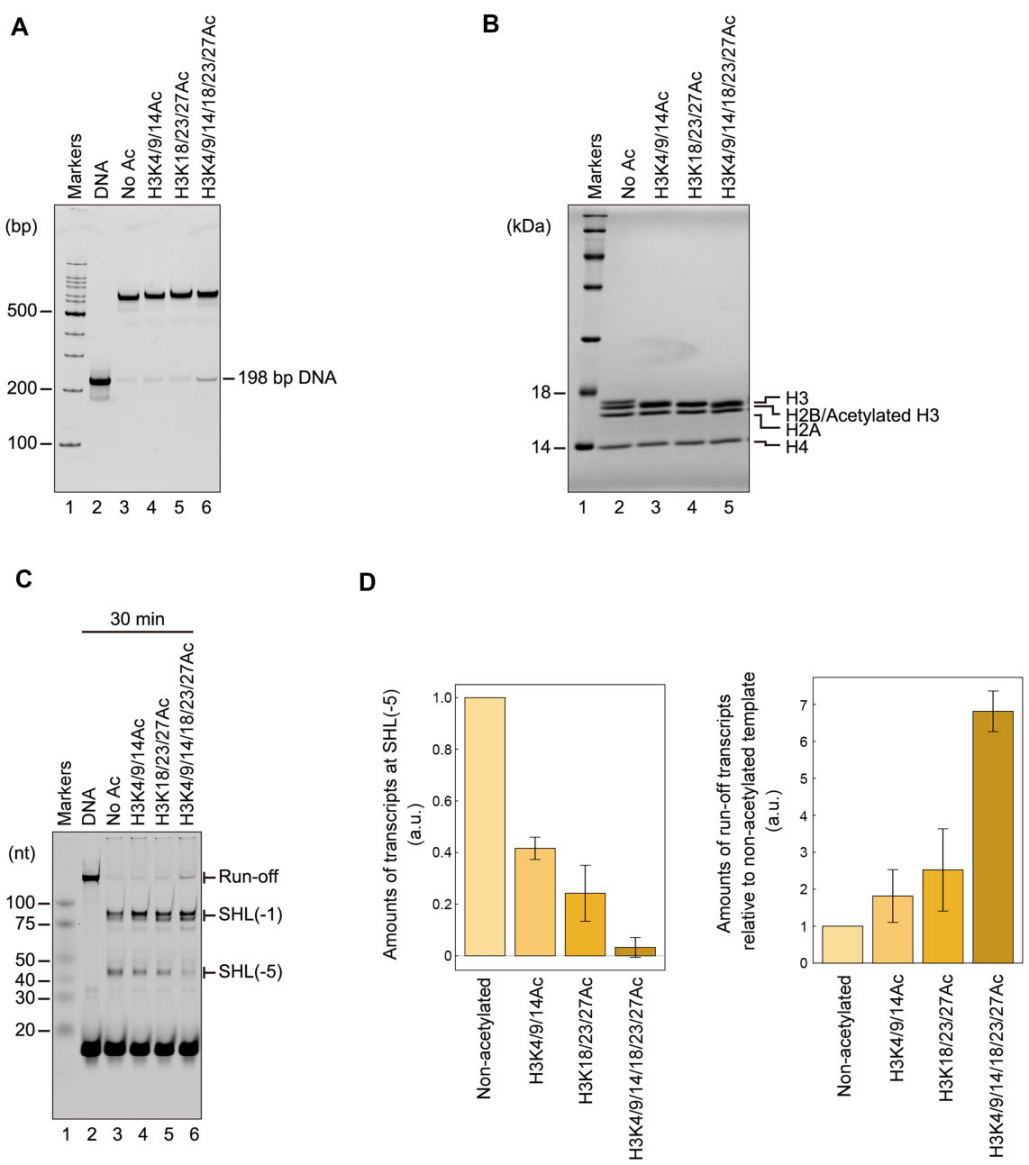
*In vitro* transcription assays of nucleosomes containing acetylated H3 tails. (**A**) The non-acetylated nucleosome and acetylated nucleosomes containing the H3K4/9/14Ac, H3K18/23/27Ac, or H3K4/9/14/18/23/27Ac peptide were analyzed by nondenaturing-PAGE with ethidium bromide staining. (**B**) Histone compositions of the non-acetylated nucleosome and acetylated nucleosomes containing the H3K4/9/14Ac, H3K18/23/27Ac, or H3K4/9/14/18/23/27Ac peptide were analyzed by SDS-PAGE with CBB staining. (**C**) Transcription assays of the 198 bp DNA, the non-acetylated nucleosome, and acetylated nucleosomes containing the H3K4/9/14Ac, H3K18/23/27Ac or H3K4/9/14/18/23/27Ac peptide. RNA transcripts were analyzed by denaturing-PAGE and detected by DY647 fluorescence. The reaction was conducted for 30 min. The transcription experiments were repeated three times, and the reproducibility was confirmed (Supplementary Figure S6). (**D**) Quantification of the RNA transcripts. Amounts of the SHL(–5) RNA transcripts of the acetylated nucleosome templates were estimated relative to the non-acetylated nucleosome template (a.u.) (left). Amounts of the run-off RNA transcripts relative to the non-acetylated nucleosome template were estimated relative to the non-acetylated nucleosome template (a.u.) (right). Quantitative data are displayed as mean value ± SD (*n* = 3 independent replicates).

In addition to H3K4/9/14/18/23/27Ac, we prepared full-length H3 peptides, H3K4/9/14Ac and H3K18/23/27Ac, in which acetyl-lysine residues were inserted at the Lys4, 9 and 14 sites, and the Lys18, 23 and 27 sites, respectively (Figures 3A and 4A and B). We then performed the RNAPII transcription assay with the nucleosomes containing each of the H3K4/9/14Ac, H3K18/23/27Ac, and H3K4/9/14/18/23/27Ac peptides (Figure 4A and B). The RNAPII pausing at the SHL(–5) position was drastically decreased in the H3K4/9/14/18/23/27Ac nucleosome, and the amount of the run-off transcript was substantially increased (Figure 4C, lane 6, and D). Similarly, in both the H3K4/9/14Ac and H3K18/23/27Ac nucleosomes, the SHL(-5) pausing was clearly alleviated, and the amount of the run-off transcript was concomitantly increased (Figure 4C and D). These results indicated that the acetylation of the H3 N-terminal tail enhances the RNAPII transcription on the nucleosome, probably by alleviating the RNAPII pausing at the SHL(–5) position. The H3 N-terminal tails protrude from the nucleosome core and are located near the entry/exit region of the nucleosomal DNA, and also bind to the nucleosomal and linker DNAs (6,13). Therefore, the acetylation of the H3 N-terminal tail may reduce the nucleosomal DNA binding capability and directly up-regulate the nucleosome transcription elongation by RNAPII.

## DISCUSSION

To elucidate the contributions of histone tails in nucleosome transcription, we studied the functions of RNAPII with the N-terminally tail-less histones H2A, H2B, H3, and H4. A cryo-EM analysis confirmed that the deletion of these histone tails did not alter the nucleosome structure (Figure 1D). Our nucleosome transcription assay revealed that the N-terminal tail of H3, but not H2A, H2B, and H4, substantially reduces the RNAPII pausing at the SHL(–5) position (Figure 2E). Interestingly, clipped H3 N-terminal tails are reportedly detected in mammalian sperm and cells (47–52). In light of the enhanced RNAPII transcription in the nucleosome containing tlH3, the biological clipping of the H3 N-terminal tail may have a specific function to alleviate the nucleosome barrier during RNAPII transcription in certain stages of cell growth.

Cathepsin L is a protease that removes 21 amino acid residues from the H3 N-terminal tail, and reportedly functions during stem cell differentiation in mice (48). Cathepsin G can also remove 19 amino acid residues from the H3 N-terminal tail (49). Recently, MMP-2 has been identified as an H3 N-terminal protease that removes 18 amino acid residues (50). MMP-2 reportedly accumulates around the + 1 nucleosome just downstream of transcription start sites, positively correlates with gene expression, and promotes myoblast differentiation (50,51). The + 1 nucleosome is considered to play an important role in transcriptional regulation (53). Intriguingly, on the +1 nucleosome, the SHL(-5) position is the major RNAPII pausing site (19). This suggests that the H3 tail clipping by MMP-2 may alleviate the transcription barrier at the +1 nucleosome during the initial stage of transcription elongation. Another protease, MMP-9, which removes the H3 N-terminal 18 amino acid residues, also reportedly promotes gene activation during osteoclastogenesis (52). The H3 N-terminal tail clipping induced by these proteases may directly enhance the nucleosome transcription by RNAPII during these biological processes.

The transcription enhancement by the H3 N-terminal tail deletion prompted us to test the effects of H3 N-terminal tail acetylation in nucleosome transcription. This acetylation substantially enhanced the transcription elongation by RNAPII in the nucleosome (Figure 4). A solution NMR analysis revealed that the H3 N-terminal tail acetylation exerts minor effects on the nucleosome core dynamics (16). Consistently, our cryo-EM analysis showed that the H3 N-terminal tail acetylation did not alter the nucleosome core structure (Figure 3D). How does the acetylation of the H3 N-terminal tail alleviate the nucleosomal inhibition of RNAPII transcription? Previous NMR analyses also demonstrated that, in the nucleosome, the acetylation of each histone tail increases its dynamics in solution (14–16). Congruent with these NMR observations, the acetylation of the H3 N-terminal tail reportedly augments the flexibility of the linker DNA regions of the nucleosome, as revealed by a FRET analysis (17). The enhanced flexibility of the linker DNA by the H3 acetylation may stimulate the local RNAPII processivity, and affect the SHL(–5) pausing (Figure 4). Interestingly, when it competes with linker histone H1 binding, the H3 N-terminal tail bound to the linker DNA is relocated to the DNA region directly bound to the histone octamer (13). The RNAPII progression to the linker DNA may also competitively remove the H3 N-terminal tail bound to the linker DNA, and thus promote its relocation to the nucleosomal DNA. In this manner, the H3 N-terminal tail could augment the RNAPII pausing at the SHL(–5) position. Further studies are awaited.

In the present study, we found that the acetylation of the H3 N-terminal tail alleviates the nucleosome barrier for transcription elongation by RNAPII in the nucleosome. The transcription coactivator p300 is a major ‘writer’ acetyltransferase for nucleosomal histones (54). p300 binds to the nucleosomal DNA in various conformations and acetylates multiple histone tails, including the H3 N-terminal tail, in the nucleosome (54–56). In certain types of cancer cells, dysregulation of p300 induces the improper expression of tumor suppressor genes and oncogenes (57). These findings suggest that histone acetylation by p300 may play an essential role in proper gene expression in cells. In good agreement with this, p300 reportedly enhances transcription in chromatinized DNA (58). It will be intriguing to study the mechanism by which the p300-mediated histone acetylation directly stimulates RNAPII transcription, through the acetylation-dependent alleviation of histone tail-DNA binding in chromatin.

## Supplementary Material

gkad754_Supplemental_File

## Data Availability

Atomic coordinates and cryo-EM maps have been deposited in the PDB and EMDB under accession numbers 8JLA and EMD-36390 (the tail-less nucleosome), 8JL9 and EMD-36389 (the canonical nucleosome), 8JLD and EMD-36393 (the H3 acetylated nucleosome), and 8JLB and EMD-36391 (the H3.2 C110A nucleosome), respectively.
