# Supplementary material for: Contributions of histone tail clipping and acetylation in nucleosome transcription by RNA polymerase II
Source: Nucleic Acids Res. 2023 Sep 18;51(19):10364–74. doi: 10.1093/nar/gkad754 (PMC10602921; doi:10.1093/nar/gkad754)
Supplement: gkad754_Supplemental_File [file gkad754_supplemental_file.pdf]

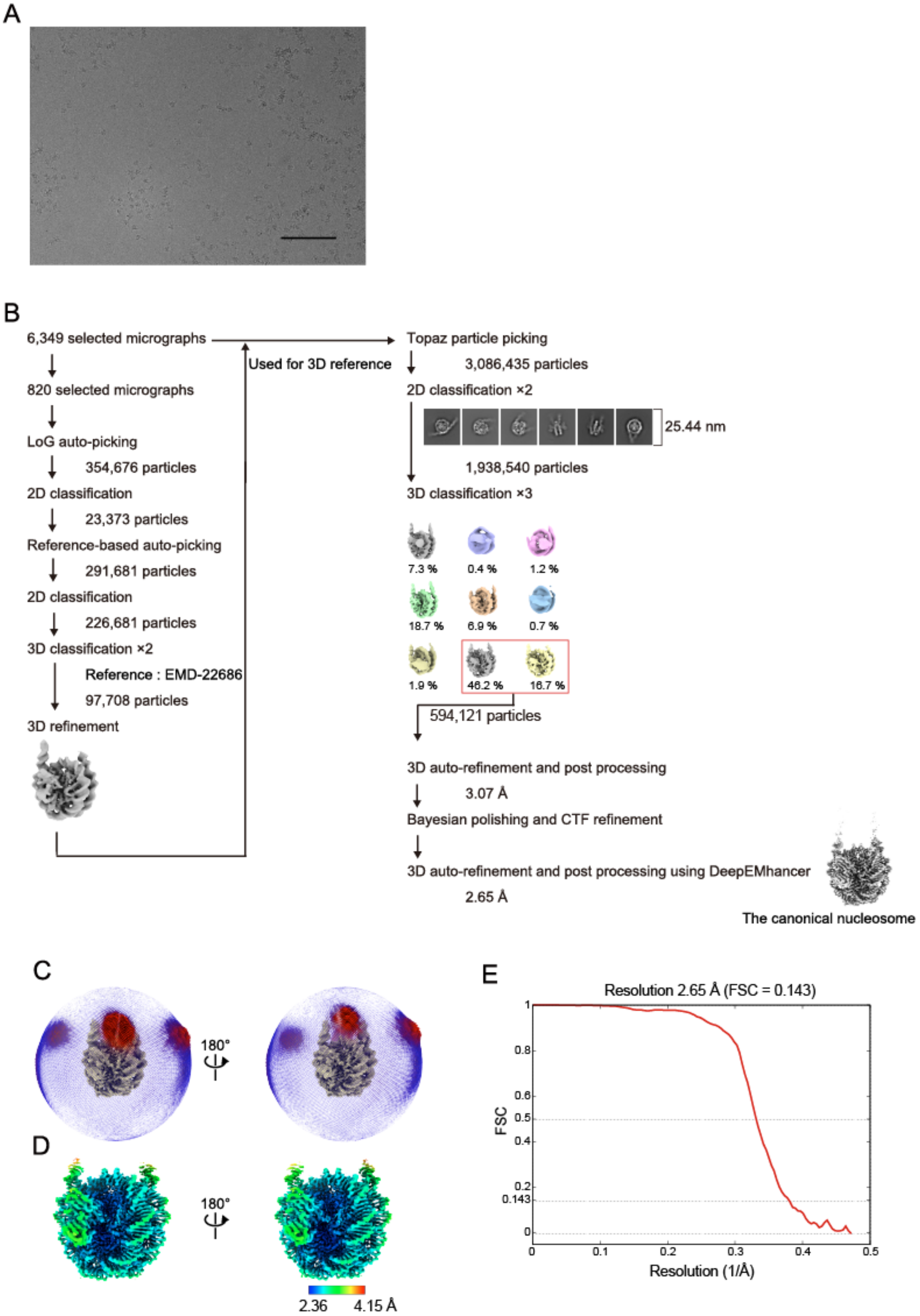

Supplementary Figure S1. Cryo-EM analysis of the canonical nucleosome. (A) Representative micrograph of the

canonical nucleosome with PL2-6 scFv. Scale bar indicates 100 nm. (B) Workflow for the canonical nucleosome with PL2-6 scFv. (C) Euler angular distribution, (D) local resolution map, and (E) Fourier Shell Correlation (FSC) curve of the canonical nucleosome with PL2-6 scFv. The resolution of this canonical nucleosome was estimated to be 2.65 Å (FSC = 0.143).

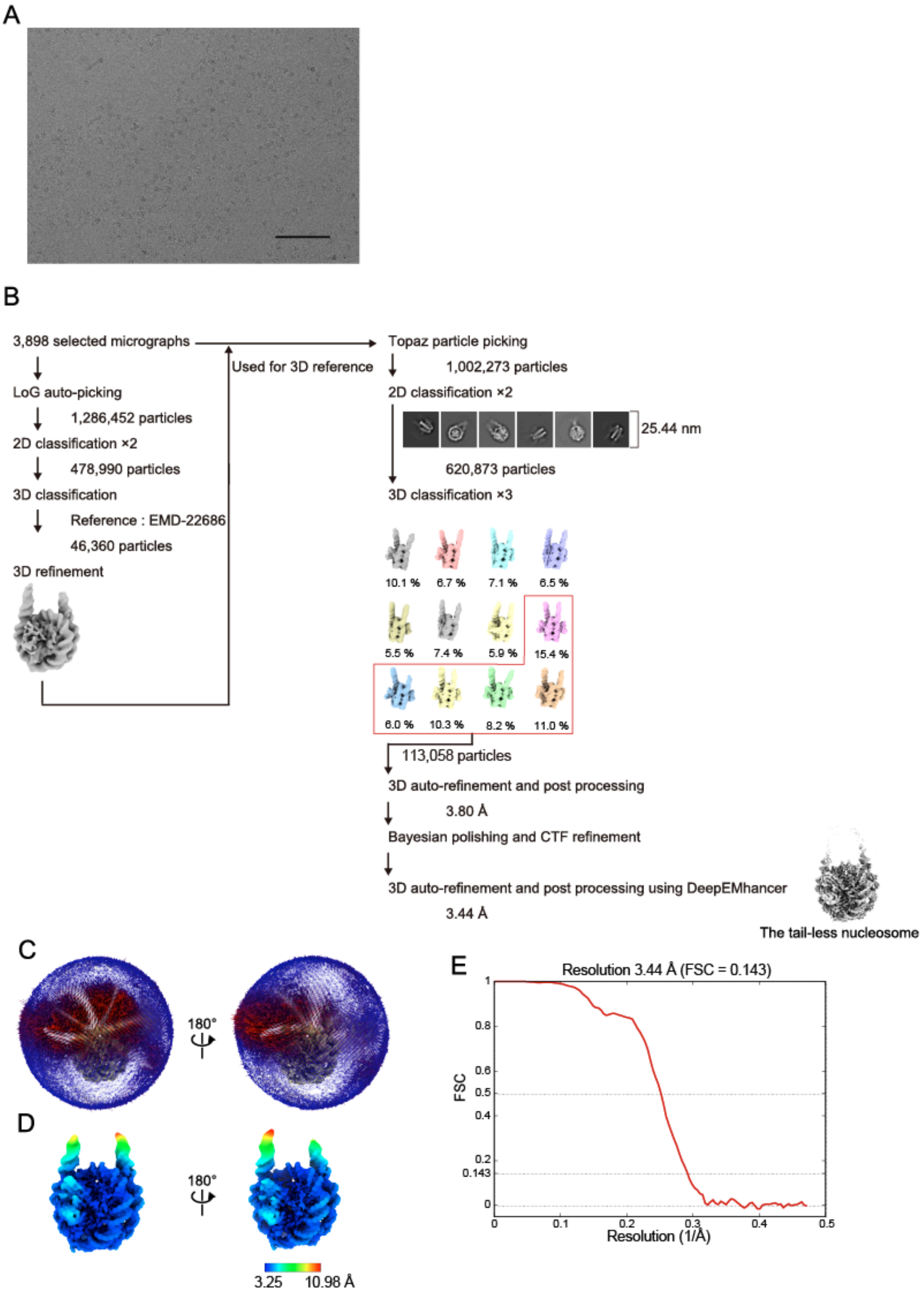

Supplementary Figure S2. Cryo-EM analysis of the nucleosome containing tail-less histones. (A) Representative micrograph of the tail-less nucleosome with PL2-6 scFv. Scale bar indicates 100 nm. (B) Workflow for the tail-less

nucleosome with PL2-6 scFv. (C) Euler angular distribution, (D) local resolution map, and (E) FSC curve of the tail-less nucleosome with PL2-6 scFv. The resolution of this nucleosome was estimated to be 3.44 Å (FSC = 0.143).

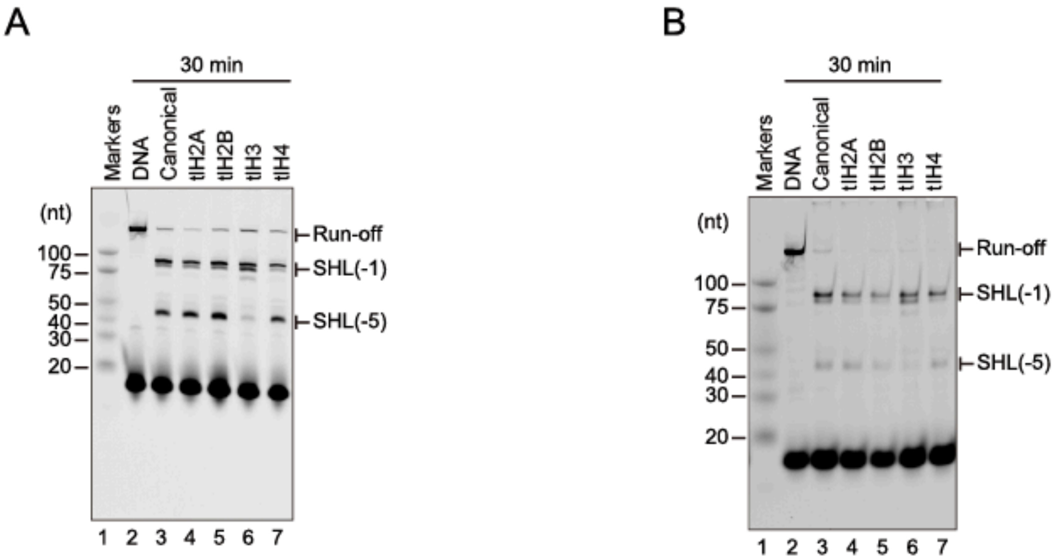

Supplementary Figure S3. Repeated *in vitro* transcription assay of the tail-less nucleosomes, as shown in Figure 2 (E).

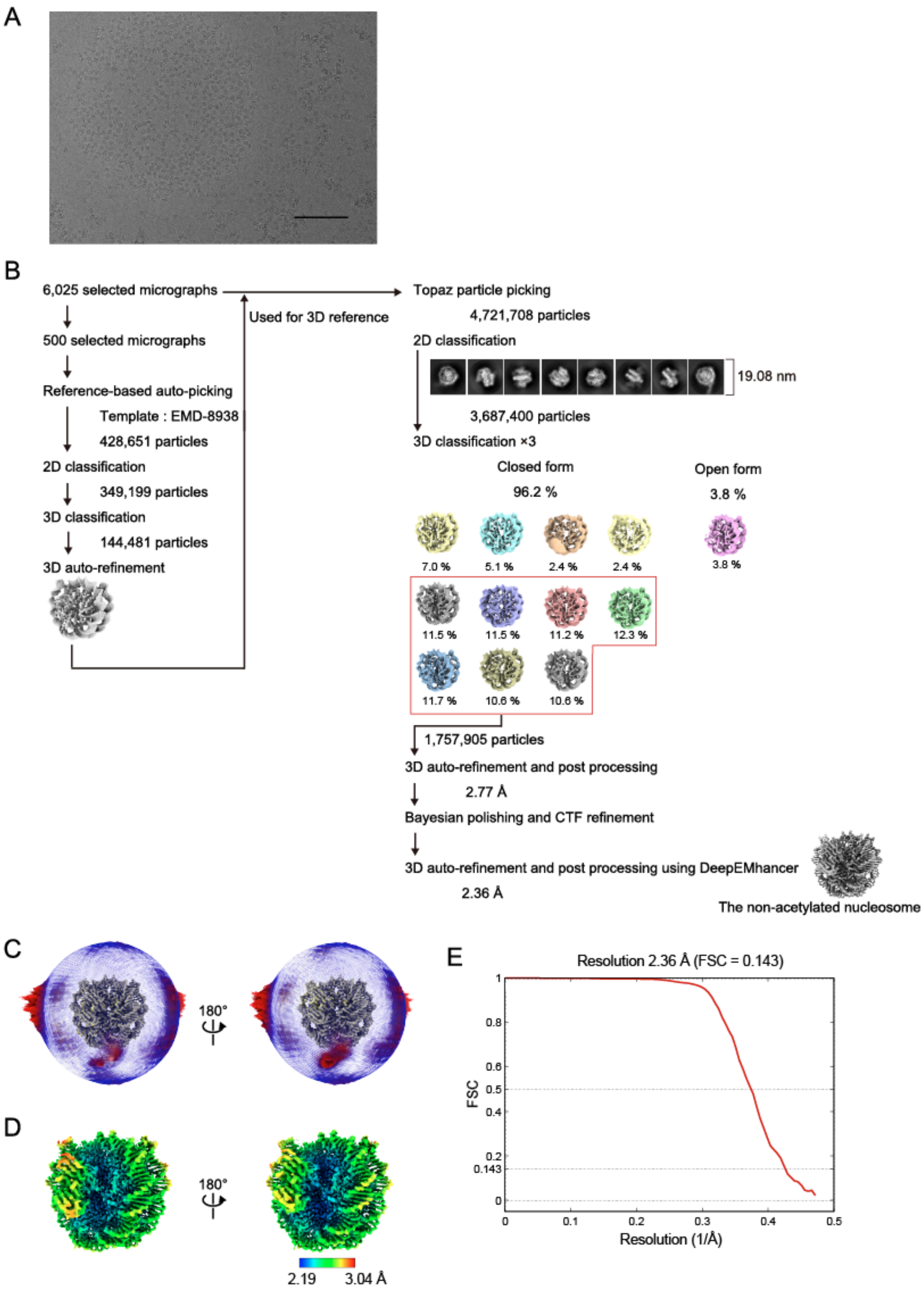

Supplementary Figure S4. Cryo-EM analysis of the non-acetylated nucleosome. (A) Representative micrograph of the non-acetylated nucleosome with PL2-6 scFv. Scale bar indicates 100 nm. (B) Workflow for the non-acetylated

nucleosome with PL2-6 scFv. (C) Euler angular distribution, (D) local resolution map, and (E) FSC curve of the non-acetylated nucleosome with PL2-6 scFv. The resolution of this nucleosome was estimated to be 2.36 Å (FSC = 0.143).

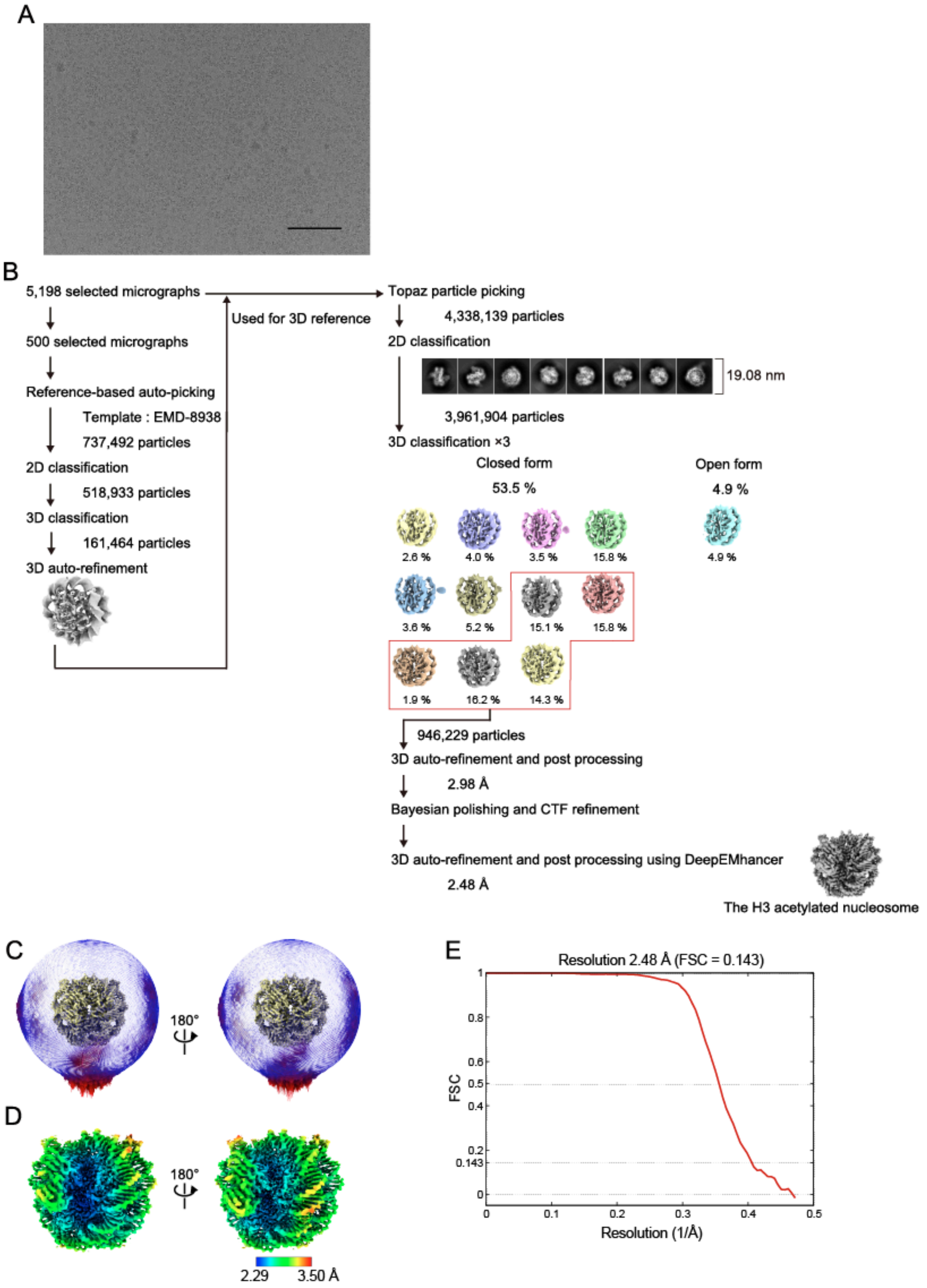

Supplementary Figure S5. Cryo-EM analysis of the nucleosome containing the H3K4/9/14/18/23/27Ac peptide with PL2-6 scFv. (A) Representative micrograph of the nucleosome containing the H3K4/9/14/18/23/27Ac peptide with PL2-

6 scFv. Scale bar indicates 100 nm. (B) Workflow for the nucleosome containing the H3K4/9/14/18/23/27Ac peptide with PL2-6 scFv. (C) Euler angular distribution, (D) local resolution map, and (E) FSC curve of the nucleosome containing the H3K4/9/14/18/23/27Ac peptide with PL2-6 scFv. The resolution of this nucleosome was estimated to be 2.48 Å (FSC = 0.143).

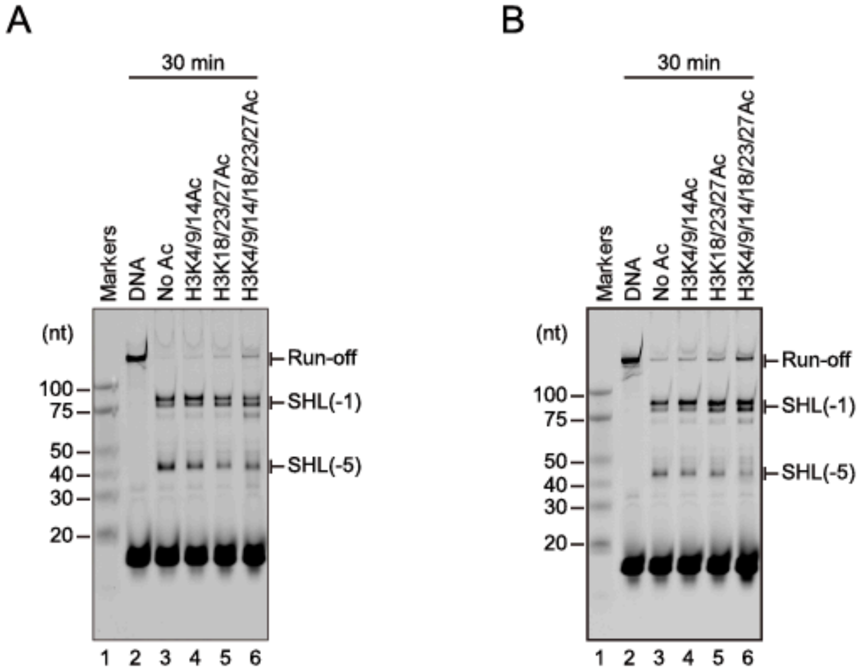

Supplementary Figure S6. Repeated *in vitro* transcription assay of nucleosomes containing acetylated H3 peptides, as shown in Figure 4 (C).
